# LigB subunit vaccine confers sterile immunity against challenge in the hamster model of leptospirosis

**DOI:** 10.1371/journal.pntd.0005441

**Published:** 2017-03-16

**Authors:** Neida L. Conrad, Flávia W. Cruz McBride, Jéssica D. Souza, Marcelle M. Silveira, Samuel Félix, Karla S. Mendonça, Cleiton S. Santos, Daniel A. Athanazio, Marco A. Medeiros, Mitermayer G. Reis, Odir A. Dellagostin, Alan J. A. McBride

**Affiliations:** 1 Biotechnology Unit, Technological Development Centre, Federal University of Pelotas, Pelotas, Rio Grande do Sul, Brazil; 2 Life Science and Health Centre, Catholic University of Pelotas, Pelotas, Rio Grande do Sul, Brazil; 3 Faculty of Veterinary Science, Federal University of Pelotas, Pelotas, Rio Grande do Sul, Brazil; 4 Gonçalo Moniz Institute, Oswaldo Cruz Foundation, Ministry of Health, Salvador, Bahia, Brazil; 5 Faculty of Medicine, Federal University of Bahia, Salvador, Bahia, Brazil; 6 Bio-Manguinhos, Oswaldo Cruz Foundation, Rio de Janeiro, Rio de Janeiro, Brazil; Centers for Disease Control and Prevention, UNITED STATES

## Abstract

Neglected tropical diseases, including zoonoses such as leptospirosis, have a major impact on rural and poor urban communities, particularly in developing countries. This has led to major investment in antipoverty vaccines that focus on diseases that influence public health and thereby productivity. While the true, global, impact of leptospirosis is unknown due to the lack of adequate laboratory diagnosis, the WHO estimates that incidence has doubled over the last 15 years to over 1 million cases that require hospitalization every year. Leptospirosis is caused by pathogenic *Leptospira* spp. and is spread through direct contact with infected animals, their urine or contaminated water and soil. Inactivated leptospirosis vaccines, or bacterins, are approved in only a handful of countries due to the lack of heterologous protection (there are > 250 pathogenic *Leptospira* serovars) and the serious side-effects associated with vaccination. Currently, research has focused on recombinant vaccines, a possible solution to these problems. However, due to a lack of standardised animal models, rigorous statistical analysis and poor reproducibility, this approach has met with limited success. We evaluated a subunit vaccine preparation, based on a conserved region of the leptospiral immunoglobulin-like B protein (LigB(131–645)) and aluminium hydroxide (AH), in the hamster model of leptospirosis. The vaccine conferred significant protection (80.0–100%, *P* < 0.05) against mortality in vaccinated animals in seven independent experiments. The efficacy of the LigB(131–645)/AH vaccine ranged from 87.5–100% and we observed sterile immunity (87.5–100%) among the vaccinated survivors. Significant levels of IgM and IgG were induced among vaccinated animals, although they did not correlate with immunity. A mixed IgG1/IgG2 subclass profile was associated with the subunit vaccine, compared to the predominant IgG2 profile seen in bacterin vaccinated hamsters. These findings suggest that LigB(131–645) is a vaccine candidate against leptospirosis with potential ramifications to public and veterinary health.

## Author summary

Leptospirosis, also known as Weil’s disease, is spread by contact with infected animals or with water and soil containing pathogenic spirochaetes belonging to the *Leptospira* genus. Leptospirosis is a serious public health problem that can cause kidney failure, pulmonary complications and can be fatal. Due to its similarity to other tropical fevers, leptospirosis is difficult to diagnose. It occurs mainly in developing countries with tropical climates and the WHO considers it one of the most widespread zoonotic diseases in the world. Existing vaccines, known as bacterins, are not recommended for general use and cause serious side-effects. Advances in the field of leptospirosis research have identified leptospiral proteins for use in a recombinant vaccine. However, evaluations using animal models reported mixed success and this has raised doubts as to their usefulness. The current study reports, for the first time, the evaluation of a subunit vaccine that reproducibly protected hamsters against leptospirosis and that induced sterile immunity among survivors. Significant antibody levels were induced in vaccinated animals and the antibody profile was characterised and found to be different to that induced by a bacterin vaccine. These observations suggest that we have identified a potential vaccine candidate for human an animal leptospirosis.

## Introduction

Leptospirosis, a spirochaetal zoonosis, has spread from its traditional rural base to cause epidemics in the urban centres of developing countries. Outbreaks occur during seasonal periods of heavy rainfall and affect at-risk groups in poor, urban slum communities. However, the global burden is under-estimated since most countries cannot perform the standard laboratory diagnostic tests required for surveillance. Surveys carried out by the World Health Organization, estimated the annual global burden to be over 1 million severe cases, resulting in 60,000 deaths [1]. Efforts to identify and control environmental sources of transmission are complicated by the fact that pathogenic *Leptospira* spp. can survive in soil and water and retain infectivity for more than one month [2, 3]. Rural leptospirosis is considered difficult, if not impossible, to control because of the broad spectrum of animal reservoirs and continuous transmission between sylvatic and domestic hosts. In the urban setting, interventions have targeted the domestic rat reservoir. However, the high density of rats makes chemical and ecological interventions ineffective. Brazil has a population of over 200 million individuals, 8% of which live in urban slums (*favelas*) [4]. While poverty levels have improved, over 10 million Brazilians subsist on less than US$2/day [5]. Currently, the prevention of leptospirosis through the discovery of novel vaccines candidates is a major research focus due to the lack of effective control measures.

Most leptospiral vaccine research has been directed toward veterinary applications [6]. Yet despite widespread vaccination, leptospirosis remains prevalent in domestic cattle, pigs, and dogs [7], which serve as reservoirs for human infection. The efficacy of whole-cell (bacterin) vaccines depends on the LPS component; active immunization with LPS and passive immunization with monoclonal antibodies specific for LPS are also effective [8–10]. Several problems with the current bacterin vaccines limit their use in humans: they induce only short-term immunity and are associated with serious side-effects. The variability of the leptospiral LPS carbohydrate antigen accounts for the serovar specificity of LPS-based vaccines and there is little or no cross-protection against infection with other leptospiral serovars, reviewed in [6].

Outer membrane proteins (OMPs) are attractive alternatives to LPS-based vaccines because of their antigenic conservation among *Leptospira* spp. and serovars. Several OMPs are surface-exposed and expressed during infection of the mammalian host [11]. When produced as recombinant proteins, the porin OmpL1 and the lipoproteins LipL41 and LipL32 were not immunoprotective [12, 13]. However, when expressed as membrane proteins in *E*. *coli*, OmpL1 and LipL41 exhibited synergistic protection in the hamster model of leptospirosis [13]. In a gerbil model of acute leptospirosis, an adenovirus construct encoding LipL32 protected 87% of vaccinated animals compared to 51% of the control group [12]. In addition, immunization of gerbils with a *lipL32* DNA vaccine provided partial protection against lethal challenge [14].

The genes encoding the leptospiral immunoglobulin-like (Lig) proteins were originally discovered by screening bacteriophage lambda expression libraries with human and equine leptospirosis sera [15, 16]. The Lig proteins belong to a family of bacterial immunoglobulin-like (Big) domain proteins that includes intimin and invasin from enteropathogenic *E*. *coli* and *Yersinia* spp., respectively [17, 18]. Three Lig proteins have been described, designated LigA, LigB, and LigC [15]. LigA contains 13 Big domains, while LigB and LigC contain 12 Big domains followed by large carboxy-terminal domains. Virulent forms of *L*. *interrogans* serovar Copenhageni strain Fiocruz L1-130 and *L*. *kirschneri* serovar Grippotyphosa strain RM52 express LigA and LigB with sequence-identical N-terminal regions, while in both strains the locus encoding LigC is a pseudogene [15]. A mouse-adapted strain of *L*. *interrogans* serovar Manilae expressed LigA and a C-terminal truncated LigB [19]. Immunization of C3H-HeJ mice with either Lig protein was found to protect against lethal challenge. However, mice are considerably less susceptible to leptospiral challenge than either hamsters or gerbils and are not an ideal model of leptospirosis [20]. Hamsters immunized with a recombinant LigA vaccine and challenged with *L*. *interrogans* serovar Pomona were protected [21]. However, 57–88% of control animals survived, indicating that the challenge strain was poorly virulent. Nevertheless, the C-terminal portion of LigA in a vaccine preparation containing Freund’s adjuvant (FA), was immunoprotective in the hamster model [22].

In the present study, we produced a recombinant protein fragment based on the N-terminal region of LigB, rLigB(131–645), also known as LigBrep, that was adsorbed to an aluminium hydroxide (AH) adjuvant. We demonstrated that a rLigB(131–645)/AH vaccine preparation conferred sterile immunity against lethal challenge in the hamster model of acute leptospirosis and that the observed immunoprotection was due to an antibody-dependent response mechanism.

## Materials and methods

### Ethics statement

All animal experimentation was conducted following the Brazilian Guide for the Production, Maintenance and Use of Animals for Teaching Activities and Scientific Research, adhering to international guidelines. All protocols were reviewed and approved by the Ethics Committee on Animal Experimentation (CEEA No. 3782–2012) at the Federal University of Pelotas (UFPel). The CEEA at UFPel is accredited by the Brazilian National Council for Animal Experimentation Control (CONCEA). The hamsters used in the current study were provided by the animal unit at UFPel.

### Bacterial strains and culture conditions

A pathogenic strain of *L*. *interrogans*, originally isolated from a patient during an epidemic of leptospirosis in the city of Salvador, Brazil [23], and kindly provided by Dr. Albert Ko (GMI, Fiocruz), was used in the current study. A virulent isolate of *L*. *interrogans* serogroup Icterohaemorrhagiae serovar Copenhageni strain Fiocruz L1-130 was cultured in liquid EMJH (Difco, BD, São Paulo, SP, Brazil) at 30°C, as described previously [22]. Seed lots were stored in liquid nitrogen, thawed and passaged up to three times *in vitro* prior to use. Leptospires were counted in a Petroff-Hauser counting chamber (Fisher Scientific, São Paulo, SP, Brazil), using a dark-field microscope (Olympus, São Paulo, SP, Brazil).

### Cloning, expression and purification of recombinant 6× His tagged LigB protein

A recombinant protein fragment, (rLigB(131–645), from LigB (AAS69085) was selected for evaluation as a vaccine candidate in combination with the adjuvant aluminium hydroxide (Sigma-Aldrich, São Paulo, SP, Brazil). LigB(131–645), corresponding to nucleotides 391–1948 of *ligB*, was cloned, expressed and purified as described [22], with the following modifications. After induction of expression with IPTG, the *E*. *coli* BL21 Star (DE3) (Invitrogen, São Paulo, SP, Brazil) cells were harvested and lysed in equilibration buffer (8 M urea, 0.5 M NaCl, 20 mM Tris, 1 mM EDTA, 10 mM imidazole, pH 8.0) (Sigma-Aldrich), overnight at room temperature followed by centrifugation (10,000× g, 1 h, 4°C). The supernatant was applied to a nickel-charged HisTrap FF column (GE Healthcare, São Paulo, SP, Brazil) and rLigB(131–645) was purified by immobilized metal affinity chromatography (IMAC) using an automated system (AKTA Start, GE Healthcare). Bound, His-tagged proteins were washed with 15 column volumes of equilibration buffer and subsequently eluted over a 20 ml gradient using elution buffer (8 M urea, 0.5 M NaCl, 20 mM Tris, 1 mM EDTA, 500 mM imidazole, pH 8.0). The eluted rLigB(131–645) was dialyzed against PBS at 4°C for 24 h and stored at -80°C or 4°C.

### Gel electrophoresis and immunoblotting

Proteins were resolved by one-dimensional sodium dodecyl sulphate-polyacrylamide gel electrophoresis (SDS-PAGE) as described in [24]. Immunoblotting was carried out as described previously [22]. Briefly, proteins were transferred to a nitrocellulose membrane, incubated with an anti-His antibody conjugated to horseradish peroxidase (Sigma-Aldrich). The blots were revealed using DAB (Sigma-Aldrich), per the manufacturer’s protocol.

### Vaccine formulation

Protein concentrations were determined by the BCA method, per the manufacturer’s instructions (Pierce, São Paulo, SP, Brazil). The vaccine was prepared as a proportion of 200 μg of protein and 2 mg of AH adjuvant. The formulation was mixed with gentle agitation for 4 h at 4°C, aliquoted and stored at 4°C until use.

### Hamster model of acute leptospirosis

Male and female Syrian hamsters (*Mesocricetus auratus*) were used as the animal model for acute leptospirosis. The challenge dose was determined using 9-week-old hamsters and a virulent isolate of *L*. *interrogans* serovar Copenhageni strain Fiocruz L1-130, as described previously [22], with the following modifications. Groups of hamsters (n = 3) were challenged with 10^0^–10^5^ leptospires in 1 ml PBS, administered by intraperitoneal (IP) injection. Hamsters were monitored three times daily for clinical signs of leptospirosis over a period of 28 days. Endpoint criteria included: ≥10% weight loss, nasal bleeding, prostration and failure to respond to stimulation [25]. Animals that fulfilled any of the endpoint criteria were euthanized by CO_2_ narcosis. The endpoint dose (ED) that caused endpoint criteria in 50% of infected animals (ED_50_) was calculated as described previously [26].

### LigB(131–645) vaccine efficacy

Groups of 4-week-old Syrian hamsters, 10 per group (unless otherwise stated), were immunized by intramuscular (IM) injection with the vaccine preparation containing the equivalent of 20–100 μg rLigB(131–645) in 200 μl on day -28, followed by a second immunization, equivalent to 20–100 μg, on day -14. The control groups were immunized by IM injection with a preparation containing PBS and AH or with the bacterin vaccine (10^8^ heat-inactivated leptospires in 200 μl PBS). Pre-immune (PI) sera were collected by phlebotomy of the retro-orbital venous plexus two days before the first immunization and post-vaccination (PV) sera were collected on day -2. Two weeks after the second immunization (day 0), hamsters were challenged with 10× ED_50_, equivalent to 200 leptospires, by IP inoculation. The animals were monitored three times daily for endpoint criteria for up to 28 days post-challenge (PC). Animals that exhibited endpoint criteria or that survived to day 28 PC were euthanized by CO_2_ narcosis. Blood and tissue samples were collected and stored in formalin for histopathology or frozen at -80°C for molecular analysis. Kidney samples were pulverized and inoculated into EMJH medium for culture isolation as described previously [27].

### Evaluation of sub-lethal infection by quantitative PCR

Real-time quantitative PCR (qPCR) was used to detect and quantify leptospiral genomic DNA in the kidney samples collected from hamsters in the control and vaccinated groups. Genomic DNA was extracted from 25 mg of kidney tissue with the DNeasy Blood and Tissue kit per the manufacturer’s instructions (Qiagen, São Paulo, SP, Brazil). The qPCR target, *lipL32*, was cloned into the pCR2.1-TOPO vector (Invitrogen) and purified plasmid DNA was diluted to generate a standard curve ranging from 2 × 10^1^ to 2 × 10^7^ copies/reaction.

The qPCR was performed using a LightCycler 96 System (Roche Life Science, São Paulo, SP, Brazil) and each sample was assayed in triplicate. Each reaction contained 200 ng of total DNA, 0.6 μM of each primer (LipL32-f 5'-CTGAGCGAGGACACAATC and LipL32-r 5'-ATTACGGCAGGAATCCAA), 12.5 μl SYBR Green PCR Master Mix (Applied Biosystems, São Paulo, SP, Brazil) and nuclease-free water (Invitrogen) was added to a final volume of 25 μl. The qPCR protocol consisted of an initial incubation step of 95°C for 10 min, followed by 45 amplification cycles (95°C for 15 s, 58°C for 15 s and 72°C for 15 s). The LightCycler application software was used to perform an absolute quantification analysis to determine the absolute number of leptospiral genome copies/reaction, this was converted to copies/μg total DNA. When the qPCR quantitation cycle (Cq) value of a kidney sample was greater or equal to the mean Cq value of the negative controls, the sample was classified as negative for the presence of leptospiral DNA.

### Histopathology

To determine the dynamics of pathologic changes of leptospirosis in hamsters, samples were collected on days 5, 8, 9, 10 and 21 post-infection in preliminary experiments. Liver, kidney and lung tissue samples were fixed in 10% buffered formaldehyde, embedded in paraffin, and sectioned according to routine histological procedures to produce 5 μm sections that were then stained with haematoxylin and eosin as described [28]. The slides were examined in a blinded manner to prevent bias in the interpretation of the results as described previously [22]. In the challenge experiments, necropsies were performed on all animals with lethal disease and in survivors on day 28 PC. The animals in the bacterin group were examined for macroscopic alterations only.

### ELISA

An ELISA based on the rLigB(131–645) was carried out as described [22], with the following modifications. Ninety-six well microtitre plates (Polysorp, Nunc, São Paulo, SP, Brazil) were coated with 50 ng of rLigB(131–645) diluted in 50 μl of 0.1 M Na_2_CO_3_ (pH 9.6) at 4°C overnight. The wells were washed 5× with PBS-T (PBS, 0.05% Tween 20) and incubated for one hour at 37°C with 100 μl of blocking solution (PBS-T, 1% BSA). Hamster sera, diluted 1:25 in PBS-T, was added and incubated for one hour at 37°C. After 5 washes with PBS-T, anti-hamster HRP-conjugated antibody, diluted 1:500 (anti-IgM, Rockland Immunochemicals, Limerick, PA, USA) or 1:6000 (anti-IgG, Jackson ImmunoResearch, West Grove, PA, USA) or 1:4000 (anti-IgG subclasses, Southern Biotech, Birmingham, AL, USA), was added and incubated for one hour at 37°C. After 5 washes with PBS-T, 100 μl of substrate solution (10 mg ortho-phenylenodiamine (OPD, Sigma-Aldrich) in 10 ml of 0.1 M phosphate citrate buffer and 10 μl of 30% H_2_O_2_) was added to each well. The colour reaction was developed for 15 minutes and the plate was read in a microplate reader (Mindray MR-96A, São Paulo, SP, Brazil), at 450 nm. The geometric mean endpoint titres (GMTs) were determined by logarithmic regression of the reading from a serum titration to obtain a titre at the intersection with the background reading, as described previously [29].

### Imprint detection

Imprints were produced by direct contact of the longitudinally-cut surface of the kidney sample, the same region as used in the qPCR assay, onto a glass slide as described previously [30]. Briefly, the kidney imprints were dried, fixed in acetone for 3 min and incubated for 60 min with a rabbit polyclonal anti-leptospiral antibody (produced in-house) at a dilution of 1:200. After washing in PBS, the imprints were incubated with a goat anti-rabbit IgG-FITC conjugate (Sigma-Aldrich), washed in PBS and dried before visualization of stained organisms by fluorescence microscopy.

### Statistical analysis

Protection against lethal leptospirosis was evaluated by Fisher’s exact test (two-tailed) using Graphpad Prism v.6. Antibody levels were analysed with one-way ANOVA (Tukey’s multiple comparisons) to compare differences between the groups (PI, PV and PC) using Graphpad Prism v.6. For all analyses, *P*-values < 0.05 were considered significant.

## Results

### Production of rLigB(131–645)

**Fig 1:**
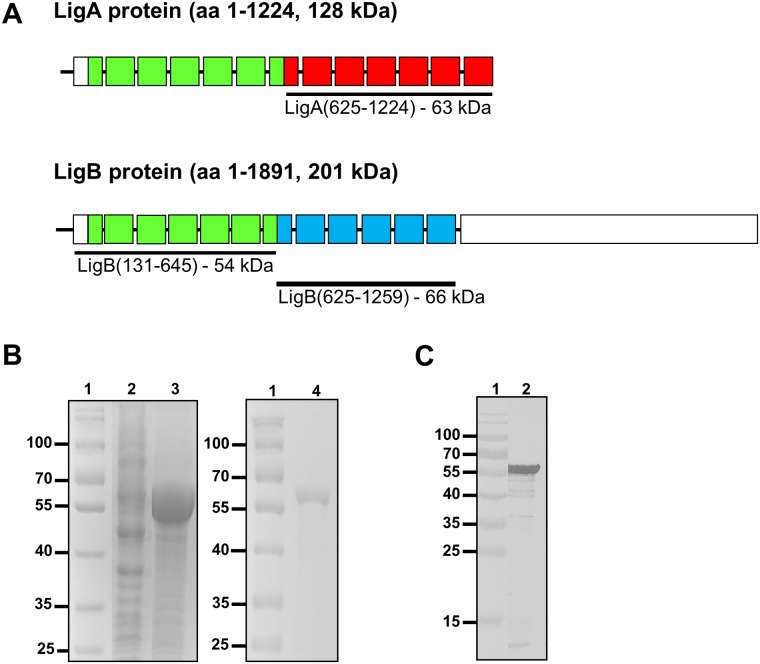
Schematic of the Lig proteins, expression and purification of rLigB(131–645). A) The full length amino acid sequences for LigA (1224 amino acids, 128.1 kDa) and LigB (1890 amino acids, 200.8 kDa) are indicated (black line), the square boxes indicate the BIDs and the LigB C-terminal domain is shown (rectangle). The recombinant proteins used as vaccine candidates are indicated: LigB(131–645) (green boxes) includes amino acids 131–645 (53.5 kDa) and is highly identical (97.9% pairwise identity) to the same region in LigA; the LigA(631–1224), also known as LigANI, (red boxes, amino acids 631–1224, 62.8 kDa) and LigB(625–1259), also known as LigBNI, (blues boxes, amino acids 625–1259, 66.2 kDa) fragments are not highly conserved (38.1% pairwise identity). B) Expression and purification of rLigB(131–645) analysed by 10% SDS-PAGE and Coomassie staining. Lanes 1: molecular mass marker (kDa); Expression of rLigB(131–645) in an *E*. *coli*(pLigB(131–645)) clone, lane 2: supernatant (soluble) fraction and lane 3: insoluble fraction; lane 4: IMAC purified rLigB(131–645), expected molecular mass of 57.2 kDa. C) Immunoblot analysis of rLigB(131–645), following transfer the nitrocellulose membrane was probed with an anti-His-HRP antibody, lane 1: molecular mass marker (kDa); lane 2: purified rLigB(131–645).

In a previous study, a vaccine preparation of recombinant LigA (rLigANI) and FA protected hamsters in a model of lethal leptospirosis [22]. However, *ligA* is only present in the genome of three out of 10 pathogenic *Leptospira* spp. [31], and is therefore not an ideal vaccine candidate. The *ligB* gene has been found in all pathogenic *Leptospira* spp. to date and therefore represents a more viable candidate [31–33]. The LigB(131–645) polypeptide used in this study included Big domains (BIDs) 2–6 and most of BIDs 1 and 7, a region that is almost identical (97.9% pairwise identity) between LigA and LigB, Fig 1A. The majority of rLigB(131–645) was expressed as an insoluble 6× His-tagged protein in *E*. *coli* and was purified by IMAC. The expected molecular mass of rLigB(131–645), including the His-tag, was 57.2 kDa, this was confirmed by SDS-PAGE and purity was estimated to be > 95%, Fig 1B. The rLigB(131–645) protein was further characterized by immunoblotting with an anti-His antibody and a single band corresponding to rLigB(131–645) with minimal degradation was observed, Fig 1C.

### Determination of the challenge dose in the hamster model

In a series of 3 experiments, groups of hamsters were infected, by IP injection, with a dose ranging from 10^0^–10^5^ leptospires. Endpoint criteria (≥ 10% weight loss, nasal bleeding, prostration and failure to respond to stimulation), were observed in all animals from day 8 to day 14 post-infection when the infective dose was ≥ 100 leptospires, S1 Fig and S1 Table. With one exception, there was a survivor in one of the groups that was infected with the highest dose. In the animals that were infected with < 100 leptospires, endpoint criteria were not observed until day 12 or later. This indicated that the ED_50_ was < 100 leptospires and when determined by logistic function, the mean ED_50_ was 18.3 ± 13 leptospires. A challenge dose of 200 leptospires, approx. 10× ED_50_, was used in this study.

### rLigB(131–645) protects hamsters against challenge

**Fig 2:**
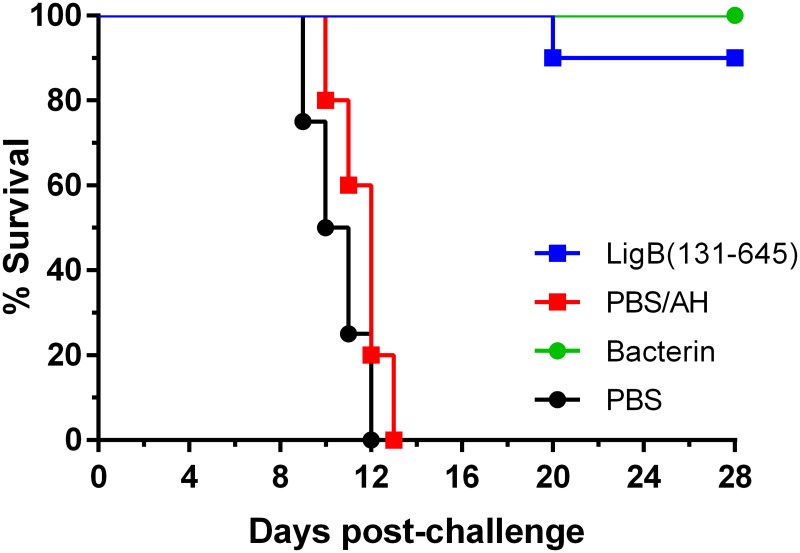
Protection against lethal challenge. Representative experiment of survival among hamsters vaccinated with rLigB(131–645), bacterin or a PBS control, followed by the administration of a potentially lethal dose of *L*. *interrogans* serovar Copenhageni strain Fiocruz L1-130, see Table 1. Groups of hamsters were immunized (days -28 and -14) with two doses (80/40 μg) of either rLigB(131–645)/AH; PBS/AH control; bacterin; or a PBS only control, and challenged with 200 leptospires (day 0). The rLigB(131–645)/AH vaccine preparation significantly protected 90.0% (9/10) of hamsters against challenge (*P* < 0.001), compared to 100% (4/4) protection in hamsters vaccinated with the bacterin (*P* < 0.05).

Seven independent experiments were performed to determine the efficacy of the rLigB(131–645)/AH vaccine formulation using two doses that ranged from 20–100 μg, see Table 1. The rLigB(131–645)/AH preparation conferred significant protection in 80.0–100% of vaccinated animals (*P* < 0.05), equivalent to a vaccine efficacy of 87.5–100%, Fig 2 and Table 1. As expected, all animals immunised with the bacterin were protected against challenge. In five independent experiments, endpoint criteria were observed in 100% of hamsters in the control group (PBS/AH), while in two experiments, 20.0 and 30.0% of the animals survived. Even though there were survivors in the negative control groups, protection was still significant in both experiments (*P* < 0.001 and < 0.05, respectively). However, this did impact on vaccine efficacy, in one experiment, efficacy was reduced to 85.7%, the lowest observed during the study. Typically, endpoint criteria were observed from days 10–18 PC, these animals were euthanized and all other surviving hamsters were euthanized on day 28. The highest dose regimen (100/100 μg) protected 80.0–90.0% of vaccinated hamsters, compared to 90.0–100% with the lower dose regimens. There was no observable dose response, even the lowest dose regimen used in the current study (20/20 μg), conferred 100% protection, Table 1.

**Table 1:** Protection conferred by immunization with rLigB(131–645) against lethal challenge in the hamster model of leptospirosis.

Vaccine	Dose (μg)	% Protection^a^		% Efficacy^b^	% Sterile immunity^c^	
		Vaccinated^d^	Control		Culture	qPCR
rLigB(131–645)	100/100	80.0 (8/10)	0 (0/4)	100	100 (8/8)	100 (8/8)
	100/100	90.0 (9/10)	30.0 (3/10)	85.7	100 (9/9)	100 (9/9)
	80/40*	90.0 (9/10)	0 (0/10)	90.0	77.8 (7/9)	ND
	80/40	100 (10/10)	0 (0/10)	100	100 (10/10)	ND
	40/20	100 (10/10)	20.0 (2/10)	100	100 (10/10)^e^	87.5 (7/8)^f^
	20/20	100 (10/10)	0 (0/10)	100	100 (10/10)	ND
	20/20	100 (10/10)	0 (0/10)	100	100 (10/10)	ND
Bacterin^g^	10^8^/10^8^*	100 (4/4)	0 (0/4)	100	100 (4/4)	75.0 (3/4)

ND—not determined.

*Survival data from these experiments are presented in Fig 2.

^a^Protection, the number of survivors/total are shown in parentheses.

^b^Efficacy is expressed as the proportionate reduction in disease attack rate between the control and vaccinated groups [34].

^c^Sterile immunity was evaluated by culture isolation and quantitative real-time PCR (qPCR). The number of sterile/total survivors are shown in parentheses.

^d^Protection was significant (*P* < 0.05) for all doses of rLigB(131–645) and bacterin.

^e^Culture isolation data not available, the presence of leptospires was determined using the imprint technique for this experiment [30, 35].

^f^Only 8/10 samples were available for qPCR analysis.

^g^The bacterin vaccine was based on two doses of 1 × 10^8^ heat-inactivated leptospires.

### rLigB(131–645) induced sterile immunity in vaccinated survivors

Previous studies have shown that while vaccine preparations based on LigANI significantly protected hamsters against challenge, sterile immunity was not induced [22, 25, 36–38]. Therefore, to characterise the protection conferred by rLigB(131–645), the surviving hamsters were evaluated for evidence of sterile immunity, Table 1. In the evaluation based on culture isolation from kidney samples, the rLigB(131–645) vaccine candidate induced sterile immunity (culture negative) in 100% of the survivors in 6/7 independent experiments. In one experiment, 77.8% (7/9) of the survivors were culture negative. The negative control groups were culture positive for all animals that developed endpoint criteria during 6/7 challenge experiments. In the remaining experiment, culture isolation data was not available, the imprint technique was used instead, and all animals that developed endpoint criteria (7/7), were positive for the presence of leptospires.

As culture isolation is not the most reliable indicator of the presence of leptospires due to their fastidious growth requirements, sterile immunity was evaluated using quantitative real-time PCR (qPCR) and the results are summarised in Table 1. All but one of the hamsters in the PBS/AH control groups were qPCR positive (24/25) and the leptospiral burden ranged from 4.3 × 10^2^ to 7.1 × 10^4^ leptospires/μg kidney DNA. In two independent experiments, 100% of the vaccinated survivors were qPCR negative, while in the remaining experiment 87.5% (7/8) of the vaccinated survivors were qPCR negative for the presence of leptospiral DNA, see Table 1. The leptospiral burden in the surviving hamster was 4.1 × 10^4^ leptospires/μg kidney DNA. The qPCR therefore confirmed the results from the culture isolation studies in all but one vaccinated survivor.

### Histopathological analysis

**Fig 3:**
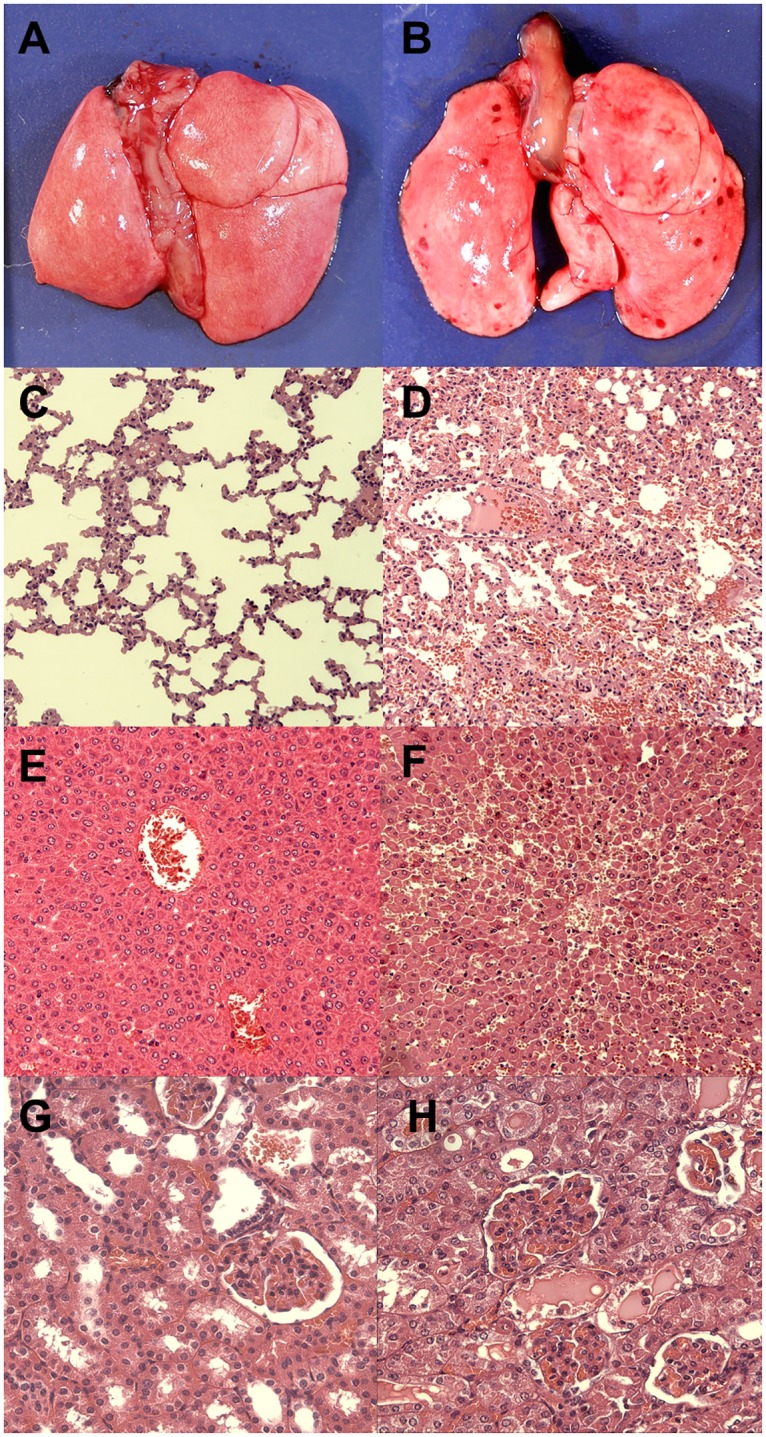
Pathological findings in the hamster model. Animals vaccinated with rLigB(131–645) (A, C, E and G) or the PBS control group (B, D, F and H) were euthanized 10 days PC and tissue samples were collected. Vaccinated animals showed no gross pulmonary lesions (A) or microscopic pulmonary lesions (C). Liver (E) and kidney samples (G) showed no evidence of microscopic abnormalities. Unvaccinated animals showed gross pulmonary haemorrhaging (B) and they were confirmed to be alveolar haemorrhages by microscopic analysis (D). Dystrabeculaton (loss of cohesion) of hepatocytes (F) and swelling of kidney tubular epithelial cells (H) were prominent features. (C-F, haematoxylin-eosin, 100× magnification and G-H, haematoxylin-eosin, 200× magnification).

Necropsies of unvaccinated hamsters and vaccinated hamsters that developed lethal disease PC showed typical features of acute leptospirosis. These findings mirrored those from preliminary experiments in hamsters with the same lethal inoculum at day 10 post-infection. Small foci of gross and microscopic pulmonary haemorrhaging were observed in the lungs of unvaccinated animals, Fig 3A and 3B. Histopathological analysis revealed diffuse dystrabeculaton of hepatocytes in unvaccinated compared to vaccinated animals. In the kidneys of unvaccinated hamsters, the most striking features included: acute swelling of the tubular epithelial cells, proteinaceous cylinders and intraglomerular haemorrhaging. The same changes were observed in vaccinated animals with lethal disease PC. No macro- or microscopic alterations were observed in the vaccinated animals that survived challenge or in survivors from the PBS control groups.

### Evaluation of the humoral immune response

**Fig 4:**
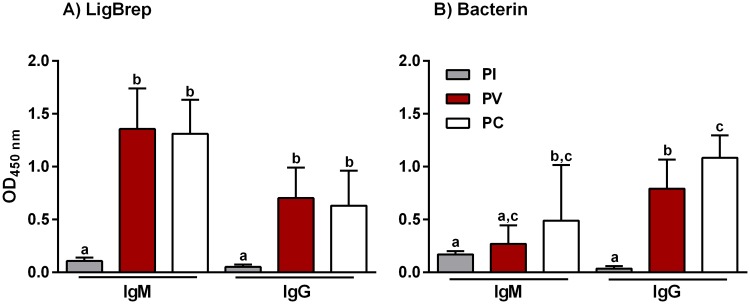
IgM and IgG induced by rLigB(131–645). ELISAs were performed to determine antibody levels in hamsters immunized with A) rLigB(131–645)/AH (80/40 μg) or B) bacterin vaccine (see Table 1). Pre-immune (PI), post-vaccination (PV) and post-challenge (PC) serum samples were collected and characterized at a single serum dilution (1:100) with anti-hamster IgM and IgG secondary antibodies. The mean optical density (OD_450 nm_) ± standard deviation (bars) from three independent experiments are shown. Significance was determined by one-way ANOVA (Tukey multiple comparison) analysis, the presence of lower case letters, where different, indicates a significant difference (*P* < 0.05) between samples.

To characterise the antibody response induced by the rLigB(131–645)/AH and the bacterin vaccine formulations, an indirect ELISA was carried out using PI, PV and PC sera and anti-hamster IgM or IgG secondary antibodies, Fig 4. Vaccination with rLigB(131–645) induced a significant IgM response PV (*P* < 0.001) and there was no significant change PC, Fig 4A. The IgM levels induced in hamsters immunized with bacterin increased PV, but only became significant PC (*P* < 0.05), Fig 4B. Hamsters immunized with rLigB(131–645) produced significant levels of IgG PV and, as observed with IgM, this did not change significantly PC, Fig 4A. In contrast, the IgG levels induced by the bacterin vaccine were significant PV and continued to increase significantly PC, Fig 4B.

When the IgG response PV was titrated, the bacterin induced the strongest IgG response with a GMT of 1:5000, compared to the highest anti-LigB(131–645) IgG GMT of 1:1450, S2 Fig. In two experiments using the 100/100 μg dose regime, the GMT was 1:1320 and 1:1445, S2A and S2B Fig, respectively, while for the 40/20 μg dose regime the GMT was 1:1450, S2C Fig. The GMTs induced by rLigB(131–645) were not dose dependent and were similar to those reported previously (1:1600) [22].

#### Non-protective antibody levels

Towards understanding why the rLigB(131–645) vaccine failed to protect some of the vaccinated animals, we analysed individual IgG antibody levels, both PV and PC. In one experiment, rLigB(131–645) failed to protect 2/10 hamsters, yet, when we compared the PV IgG levels, they were similar: in 8/10 survivors, the OD values ranged from 0.30–0.48 compared to 0.32 and 0.42 in the two hamsters that were not protected. However, when we compared the IgG levels PC, there were obvious differences. We observed previously that PC, IgG levels among survivors fell by 10–30% compared to PV levels, see e.g. Fig 4. However, in the two vaccinated hamsters that did not survive, the PC IgG levels were markedly lower: ODs of 0.02 and 0.16, respectively, a reduction of approx. 95 and 60% in each respective animal. In a separate experiment, the vaccine failed in 1/10 hamsters and we observed a similar fall in IgG levels. The hamster had one of the highest PV IgG levels, 0.92 compared to 0.62–1.08 in surviving animals, however PC, the IgG level fell by 97% to 0.03.

### Characterization of the IgG subclass profiles

**Fig 5:**
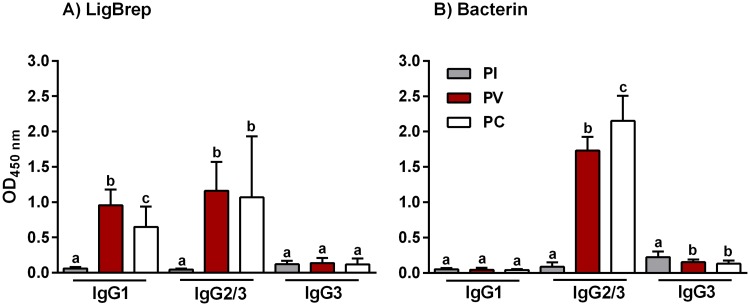
IgG subclasses induced by vaccination with LigB(131–645). IgG subclasses were characterized using ELISAs to determine antibody levels in hamsters immunized with A) rLigB(131–645)/AH or B) bacterin vaccine, see Table 1. Pre-immune (PI), post-vaccination (PV) and post-challenge (PC) serum samples were collected and characterized with anti-hamster IgG1, IgG2/3 or IgG3 conjugates. The mean OD ± standard deviation (vertical bars) from three independent experiments are shown. Significance was determined by one-way ANOVA (Tukey multiple comparison) analysis and the presence of lower case letters, where different, indicates a significant difference (*P* < 0.05) between samples.

The IgG response in vaccinated hamster was further characterised to determine the IgG subclass profiles associated with the rLigB(131–645) and bacterin vaccines, Fig 5. In hamsters vaccinated with rLigB(131–645), both IgG1 and IgG2 subclasses were significantly induced PV, Fig 5A. However, although IgG1 levels fell significantly PC, they remained significantly higher compared to PI levels and while IgG2 levels dropped PC, it was not significant, Fig 5A. In addition, rLigB(131–645) did not appear to induce IgG3 during the study. The IgG subclass profile in hamsters immunized with the bacterin vaccine was almost exclusively IgG2, both PV and PC, Fig 5B. Furthermore, there was no significant production of IgG1 and the already low PI IgG3 levels fell significantly PV and PC.

## Discussion

Towards controlling the increasing problem of leptospirosis, we have focused on identifying and screening proteins from pathogenic *Leptospira* spp. that are potential vaccine candidates. Previously, several groups have shown that hamsters immunized with recombinant polypeptides from the C-terminal region of LigA are protected against lethal challenge [22, 25, 38]. In terms of protection, LigA is probably the best vaccine candidate identified to date, reviewed in [11]. However, sub-lethal levels of infection were detected in the majority of surviving animals, regardless of the delivery route or the adjuvant used [21, 22, 25, 36, 38, 39]. Furthermore, LigA is not conserved amongst all pathogenic *Leptospira* spp., it has been identified in only three pathogenic species to date [31], while LigB has been found in all pathogenic *Leptospira* spp. [31, 33, 40]. The identity of LigB varies from approximately 64–93%, while the LigB(131–645) region is less well conserved (59.9–93.4%) among pathogenic *Leptospira* spp. with the exception of *L*. *noguchii*, see S2 Table and S3 Fig.

Koizumi and colleagues reported that recombinant proteins based on the identical and non-identical regions of LigA(68–1224) and LigB(68–1191) in a vaccine preparation with FA conferred 90–100% protection in a mouse model, although 40% of the control group survived [19]. However, an evaluation of rLigB(131–645) and rLigB(625–1259), with FA, reported that they failed to protect vaccinated hamsters [22]. In another study, the identical region of LigB(32–630) and AH protected 62–75% of vaccinated animals [41]. However, while protection was statistically significant in 2/3 experiments, none of the LigB vaccine preparations induced sterile immunity. In addition, an evaluation of a recombinant LigB(1–1890) found that immunised hamsters seroconverted but remained susceptible to infection in the hamster colonisation model [42]. We therefore concentrated our efforts towards developing a LigB vaccine candidate that could address these shortcomings.

In a preliminary study, we evaluated rLigB(131–645) and rLigB(625–1259) in vaccine preparations containing AH. The rLigB(625–1259)/AH formulation failed to protect any of the vaccinated animals against challenge (see S3 Table), in agreement with a previous report [22]. However, vaccination with rLigB(131–645)/AH protected 90% of hamsters against challenge. Furthermore, of these nine survivors, seven were culture negative, suggesting that sterile immunity may have been induced. We therefore focused on LigB(131–645) as the more promising vaccine candidate.

In an additional six experiments, the rLigB(131–645)/AH vaccine preparation conferred significant protection (80–100%, *P* < 0.05), equivalent to an efficacy of 86–100%. Furthermore, all the surviving hamsters were culture negative, indicating that the rLigB(131–645) vaccine induced sterile immunity. To confirm these observations, kidney samples were evaluated for the presence of leptospiral DNA by qPCR. In three independent experiments, 8/8, 9/9 and 7/8 vaccinated survivors were negative for the presence of leptospiral DNA. Further confirmation was provided by histological analysis of the kidneys, liver and lung tissue of vaccinated animals. There were no gross or microscopic alterations in the kidney, liver or lung tissues of vaccinated survivors. The authors believe that this is the first report of sterile immunity associated with a recombinant subunit vaccine that conferred significant protection against lethal challenge.

A limitation of the hamster model of leptospirosis is its dependence on a virulent challenge strain. There are reports of > 50% survival in the negative control group such that vaccine efficacy was adversely affected and protection was not significant, reviewed in [6, 11]. Therefore, to maintain virulence, a seed lot of the virulent isolate of the Fiocruz L1-130 strain used in the challenge experiments was prepared, stored in liquid nitrogen and subcultured a maximum of three times *in vitro*. The ED_50_ of this isolate was calculated in three independent experiments and found to be < 20 leptospires. Although there are no guidelines as to the challenge dose for evaluating a subunit vaccine against leptospirosis, the current USDA protocol for the evaluation of bacterin vaccines recommends a challenge dose of 10–10,000× LD_50_ [43]. In the current study, we used 200 leptospires, approx. 10× ED_50_, equivalent to the lowest recommended challenge dose. Furthermore, this challenge dose is likely to be more representative of a natural infection. Reports on the quantification of leptospires in surface water have varied considerably, with concentrations ranging from < 1 to 10^4^ leptospires/ml surface water [44–47]. In 5/7 experiments of the present study, endpoint criteria were observed in all the groups of animals immunized with PBS/AH. However, there were survivors in the control groups of two experiments. Nevertheless, due to rigorous experimental design, this had a minimum impact on vaccine efficacy and protection remained significant.

Protection against leptospirosis is generally accepted to be antibody-based and from previous reports, recombinant Lig proteins are recognised by sera from leptospirosis patients and from infected laboratory animals [15, 16]. Furthermore, sera from laboratory animals vaccinated with rLig proteins can recognise the native Lig proteins [22]. Previously, rLigA(625–1244) and rLigB(625–1259) were shown to induce high IgG titres in hamsters, while rLigB(131–645), although immunogenic, had the lowest antibody titres of all the rLig proteins evaluated [22]. In the current study, while the IgG levels were significant, the GMTs were over three times lower in rLigB(131–645) immunised hamsters compared to those vaccinated with the bacterin. We also noted that after challenge, only animals vaccinated with the bacterin produced significantly increased levels of IgG. Furthermore, while the PV IgG levels in rLigB(131–645) vaccinated hamsters that did not survive were significant, once challenged, the IgG levels fell by > 60%. This suggests that while protection was antibody-dependent, the PV IgG antibody titres were not related to protection and therefore could not be used as a correlate of immunity.

rLigB(131–645) was expressed as an insoluble protein that was subsequently refolded. As it is unlikely that the tertiary structure of rLigB(131–645) resembled that of the native LigB protein, the specific immune response in vaccinated animals was probably targeted to linear epitopes present in rLigB(131–645). Subunit vaccines are recognised by antigen presenting cells that activate naïve T cells, via a major histocompatibility complex (MHC) class II-peptide complex, these CD4 T cells subsequently activate B cells (presenting the same MHC-peptide complex), resulting in antibody production [48]. Syrian hamsters are known to produce IgM [49] and the IgG subclasses IgG1, IgG2 [50] and IgG3, although not all inbred strains express IgG3 [51]. We identified the IgG subclasses associated with protection and found that the bacterin induced significant levels of IgG2 compared to a mixed IgG1 and IgG2 response in hamsters immunized with rLigB(131–645). However, it is difficult to interpret this as little is known about the hamster immune response. The presence of IgG1 in the mouse is indicative of a Th2-like response and one that is most effective against extracellular pathogens. While murine IgG2a, IgG2b and IgG3 are associated with a Th1 response and protection against intracellular pathogens. Therefore, as mouse and hamster immunoglobulins are related [52], and pathogenic *Leptospira* spp. are regarded as extracellular pathogens, a protective immune response should be biased towards proliferation of Th2 cells and the presence of IgG1. Moreover, in studies of the immunoglobulins induced in hamsters infected with rabies virus [53], or with *Leishmania donovani* [54], the response was primarily IgG2. In contrast, the response in hamsters immunized with soluble proteins was mainly IgG1 [55]. Additionally, hamster IgG2 binds complement via the classical pathway, while IgG1 does not [53]. This may explain why the predominantly IgG2 response induced by the bacterin vaccine was protective but did not induce sterile immunity.

Previous studies expressed the recombinant LigB polypeptides as tagged (His or GST) proteins in *E*. *coli*, purified using affinity chromatography. However, we observed that manual IMAC purification of recombinant proteins resulted in substantial lot-lot variation (due to poorly purified proteins, precipitation and degradation) and vaccine preparations that failed to protect against challenge, see e.g. S3 Table. To overcome this limitation, we minimised rLigB(131–645) lot-lot variation using pre-packed columns and an automated chromatography system with gradient elution that resulted in > 90% purification of rLigB(131–645).

The vaccine schedule used in the present study was based on two IM doses of 20–100 μg, 14 days apart, followed by an IP challenge 14 days after the second dose. This compared to the subcutaneous administration of either 3 × 10 μg in the mouse model [19] or 2 × 50 μg in the hamster model [41]. Furthermore, we did not observe any dose related effect on reducing the amount of rLigB(131–645) used in the vaccine preparation. This was not unexpected, it is the amount of antigen adsorbed to the AH adjuvant rather than the total amount used in the vaccine preparation that is responsible for immunogenicity [56]. This is one of the benefits of AH, low quantities of antigen are immunogenic, potentially reducing production costs. As no dose effect was observed, even at the lowest dosage (20/20 μg), it may be possible to further reduce the amount of rLigB(131–645) used in the vaccine preparation. Further studies will be required to determine the minimum amount of rLigB(131–645) that does not compromise the efficacy of the vaccine.

The Lig proteins play a role in the adhesion, invasion and immune evasion of pathogenic *Leptospira* spp. [57–60]. Inhibition by antibodies capable of binding to native LigB could explain the protection conferred by vaccination with rLigB(131–645). The N-terminal region of LigB has been shown to bind plasminogen [61], Factor H (FH) and C4b-binding protein (C4BP) [62]. However, recently the LigB binding domains for FH and C4BP were shown to be located towards the C-terminal region of LigB [63]. LigB bound plasminogen can be activated to plasmin that can degrade fibrinogen and the complement proteins C3b and C5. This potentially provides an advantage to leptospires during dissemination following infection, antibodies produced by rLigB(131–645) could therefore block plasminogen binding and prevent leptospiraemia.

While we have identified a vaccine candidate that is highly conserved among all pathogenic *Leptospira* spp. studied to date and one that can induce sterile immunity, several challenges remain to be addressed. Bacterin-based vaccines induce protection but are serovar specific and targeted to leptospiral LPS, see [6]. Yet, there is still no solid evidence that a subunit vaccine for leptospirosis is capable of inducing protection against a heterologous challenge. However, one study reported that leptospiral proteins (whole-cell extracts), rather than LPS, induced heterologous protection in gerbils [10], supporting the hypothesis that a highly-conserved subunit vaccine could potentially confer cross-protection. A further challenge is to understand the mechanism of rLig vaccine-mediated immunity. As both LigA and LigB contain surface exposed moieties, are adhesins [64], and potentially help leptospires inhibit complement activation [62, 63, 65], more than one mechanism may be involved. Therefore, further understanding of the immunoprotective response to leptospirosis, whether it be humoral, cellular or both, will significantly aid the selection of epitopes and adjuvants necessary to further improve the Lig subunit vaccine.

## Supporting information

S1 Fig Determination of the ED_50_ in Syrian hamsters. Groups of hamsters (n = 3) were inoculated with a range of infective doses of *L*. *interrogans* serovar Copenhageni strain Fiocruz L1-130, see S1 Table. Three independent experiments were carried to calculate the ED_50_. The number of leptospires/infective dose and hamster survival are shown.
(TIF)

S2 Fig Endpoint IgG GMTs of the LigB(131–645) and bacterin vaccine preparations. The endpoint titres were determined for sera collected from hamsters 14 days PV with LigB(131–645), and the bacterin vaccine in an ELISA based on rLigB(131–645). A-C) serial dilution of PI and PV sera collected from hamsters immunised with rLigB(131–645) in three independent experiments. D) a representative experiment for hamsters immunized with a bacterin vaccine is shown. The standard deviation (vertical bars) for each data point is shown.
(TIF)

S3 Fig Conservation of LigB and LigB(131–645) among the pathogenic *Leptospira* spp. The paired columns represent the mean amino acid pairwise identity of LigB and LigB(131–645) from *L*. *interrogans* serovar Copenhageni strain L1-130, respectively, with the corresponding proteins from pathogenic *Leptospira* spp. The error bars represent the standard deviation from the mean.
(TIF)

S1 Table Determination of the ED_50_ for *L*. *interrogans* strain Fiocruz L1-130 in the Syrian hamster model of leptospirosis. (DOCX)

S2 Table Conservation of LigB and LigB(131–645) among the pathogenic *Leptospira* spp. (DOCX)

S3 Table Protection conferred by immunization with rLigB(625–1259) and rLigB(131–645) against lethal challenge in the hamster model of leptospirosis. (DOCX)
