# Supplementary material for: LigB subunit vaccine confers sterile immunity against challenge in the hamster model of leptospirosis
Source: PLoS Negl Trop Dis. 2017 Mar 16;11(3):e0005441. doi: 10.1371/journal.pntd.0005441 (PMC5370146; doi:10.1371/journal.pntd.0005441)
Supplement: S1 Table — (DOCX) [file pntd.0005441.s004.docx]

| **Table S1**. **Determination of the ED_50_ for *L. interrogans* strain Fiocruz L1-130 in the Syrian hamster model.** | | | | | | | |
| --- | --- | --- | --- | --- | --- | --- | --- |
| Expt. | Challenge dose/EC observed | | | | | | ED_50_ |
|  | 10^5^ | 10^4^ | 10^3^ | 10^2^ | 10^1^ | 10^0^ |  |
| 1 | 3/3 | 3/3 | 3/3 | 3/3 | 1/3 | - | 17.8 |
| 2 | - | 3/3 | 3/3 | 3/3 | 0/3 | 0/2 | 31.6 |
| 3 | - | 2/3 | 3/3 | 3/3 | 2/3 | 1/3 | 5.6 |
| Mean ± SD | | | | | | | 18.3 ± 13 |

EC – Endpoint criteria; SD – Standard deviation.
