# Supplementary material for: LigB subunit vaccine confers sterile immunity against challenge in the hamster model of leptospirosis
Source: PLoS Negl Trop Dis. 2017 Mar 16;11(3):e0005441. doi: 10.1371/journal.pntd.0005441 (PMC5370146; doi:10.1371/journal.pntd.0005441)
Supplement: S2 Table — (DOCX) [file pntd.0005441.s005.docx]

**S2 Table**. Conservation of LigB and LigB(131-645) among the pathogenic *Leptospira* spp.

| *Leptospira* spp. | % Identity^a^ | |
| --- | --- | --- |
|  | LigB | LigB(131-645) |
| *L. alexanderi* | 66.1 - 66.7 | 63.5 |
| *L. alstoni* | 64.9 | 59.9 - 67.6 |
| *L. borgpetersenii* | 64.2 - 64.6 | 61.6 - 62.5 |
| *L. interrogans* | 99.3 - 100 | 96.1 - 100 |
| *L. kirschneri* | 89.6 - 92.9 | 91.0 - 93.4 |
| *L. kmetyi* | 64.7 | 62.2 |
| *L. mayottensis* | 65.0 | 63.5 |
| *L. noguchii* | 70.8 - 76.8 | 77.5 - 79.6 |
| *L. santarosai* | 65.8 - 66.2 | 63.7 - 64.3 |
| *L. weilii* | 65.6 - 67.1 | 62.5 - 68.7 |

^a^The sequences were compared using the NCBI blastp suite, the *L. interrogans* L1-130 proteins were used as the query sequences.
