# Supplementary material for: LigB subunit vaccine confers sterile immunity against challenge in the hamster model of leptospirosis
Source: PLoS Negl Trop Dis. 2017 Mar 16;11(3):e0005441. doi: 10.1371/journal.pntd.0005441 (PMC5370146; doi:10.1371/journal.pntd.0005441)
Supplement: S3 Table — (DOCX) [file pntd.0005441.s006.docx]

| **S3 Table. Protection conferred by immunization with rLigB(625-1259) and rLigB(131-645) against lethal challenge in the hamster model of leptospirosis** | | | | |
| --- | --- | --- | --- | --- |
| Vaccine | Dose (μg) | % Protection^a^ | | % Sterile immunity^b^ |
|  |  | Vaccinated | Control |  |
| LigB(625-1259) | 80/40 | 0 (0/10) | 0 (0/10) | 0 (0/10) |
| LigB(131-645)^c^ | 100/100 | 0 (0/8) | 0 (0/8) | 0 (0/8) |
|  | 40/20 | 0 (0/8) | 0 (0/8) | 0 (0/8) |

^a^Protection, the number of survivors/total are shown in parentheses.

^b^Sterile immunity was evaluated by culture isolation from kidney samples.

^c^The rLigB(131-645) lots used in these experiments degraded prior to adsorption with AH.

rLigB(625-1259) was expressed as described previously [22], groups of hamsters (n = 10) were vaccinated with two doses of a vaccine preparation of rLigB(625-1259)/AH, rLigB(131-645)/AH or with PBS/AH. Two weeks after the final dose, the hamsters were challenged with 200 leptospires (10× ED50). Hamsters that developed endpoint criteria were euthanized and kidney samples were evaluated for the presence of leptospires by culture isolation. None of the vaccinated hamsters survived lethal challenge and all cultures were positive for the presence of leptospires.
